# The Spreading of Social Energy: How Exposure to Positive and Negative Social News Affects Behavior

**DOI:** 10.1371/journal.pone.0156062

**Published:** 2016-06-02

**Authors:** Ziqing Yao, Rongjun Yu

**Affiliations:** 1 School of Psychology, Center for Studies of Psychological Application and Key Laboratory of Mental Health and Cognitive Science of Guangdong Province, South China Normal University, Guangzhou, China; 2 Department of Psychology, National University of Singapore, Singapore, Singapore; 3 Singapore Institute for Neurotechnology (SINAPSE), Centre for Life Sciences, National University of Singapore, Singapore, Singapore; 4 Neurobiology/Aging programme, Center for Life Sciences, National University of Singapore, Singapore, Singapore; Beihang University, CHINA

## Abstract

Social news, unlike video games or TV programs, conveys real-life interactions. Theoretically, social news in which people help or harm each other and violate rules should influence both prosocial and violation behaviors. In two experiments, we demonstrated the spreading effects of social news in a social interaction context emphasizing social conventions and a nonsocial interaction context emphasizing moral norms. Across the two studies, the results showed that positive social news increased cooperation (decreased defection) but had no effect on cheating, whereas negative social news increased cheating but with no change in cooperation (or defection). We conclude that there is a spreading impact of positive social news in the conventional norm domain and of negative social news in the moral norm domain.

## Introduction

As we enter the digital era, the proliferation of digital platforms supplementing traditional sources such as television, radio, and newspapers has resulted in people spending much more time with the news than was the case a decade ago [1]. Earlier studies suggested that broadcasted news stories affected an individual’s subsequent behavior and judgments [2]. Nowadays, a news item may be spread across societies and discussed widely by people within minutes via social media platforms, which may influence human behavior and social activities [3]. To date, abundant literature has examined the influence of media content (e.g., video games, TV shows, and videos) on human behaviors [4, 5]; for example, playing violent video games has been found to cause people to behave aggressively towards other people [6, 7], decrease prosocial behaviors (such as cooperative behavior) [8], and increase physiological arousal and aggression-related thoughts and feelings [9], both in short-term and also in long-term across cultures [5], but the links among these are still inconsistent [10, 11]. Using the prisoner’s dilemma game [12], Ramos, Ferguson (13) observed no significant influence of violent TV episodes on individuals’ cooperative behavior. The same results were obtained even after controlling for mood state and exposure to violent TV programs in real life [13].

However, it must be noted that the TV episodes used in the Ramos, Ferguson (10) study were fictional, and the results demonstrated that the processing of the media depended on whether the participants understood the content to be fictional or real. As the current study focuses on social news—which is for the most part real, as well as construed by consumers to be real—there might be different results than that found in earlier studies concerning the relationship between social news and human behaviors.

An understanding of media and behavior can be informed by research showing that just observing a model can guide subsequent behaviors [14]. Cialdini manipulated whether a real environment was clean or littered to find out how people were influenced. Results showed that compared to a clean environment, participants littered more in a littered environment, especially when observing a model drop trash into a littered environment [15]. Additionally, another study by Cialdini showed that people who saw a billboard stating that so much petrified wood had been stolen from a forest that the wood was now endangered were more likely to steal it themselves, compared with controls who saw billboards that either told nothing or told there was plenty of wood [15].

This phenomenon is seen in research showing that after observing others violating social norms or rules, individuals are more likely to violate other norms or rules: this is the spreading of disorder [16]. On the other hand, knowing that other people have respect for social norms can spread prosocial behavior from one norm to another. This is termed the spreading effect of cross-norm reinforcement, and it increases the likelihood of exhibiting prosocial behavior [17]. Based on the degree of their arbitrariness, social norms can be divided into different categories such as conventional and moral norms [18]. Conventional norms refer to communal opinions in a group-specific level (what other people usually do in a given setting, e.g., prohibiting wearing gender-inappropriate clothing); moral norms, for the most part, refer to rules in a generic level (acknowledged widely within the society, e.g., prohibiting injuring other people) [19, 20]. Additionally, according to Nichols (21), moral norm violations are less context-sensitive responses whereas conventional norms are on the opposite. Can contemporary social news, delivered by various types of social media, spread as well? Social news has been proved to be vital in its contribution to forming social norms and public opinion. If so, how might it affect conventional and moral norms?

The current study builds on earlier research in two ways. First, we consider not just social interactions but also nonsocial. More specifically, it is worthy of note that most of the research to date has mainly involved social interactions, such that each person only has partial control of the outcomes [22]. However, non-social interactions such as cheating are quite different from social interactions, and cheating appears to be influenced by situational factors to some degree [23]. For the purposes of this study, cheating is defined as unethical behavior displayed in the context of a problem-solving task [24]. Cooperation is defined as prosocial behavior displayed in the prisoner’s dilemma game. Second, we take into account cognitive processes and an emotional component in decision making. Social cognitive theory holds that individuals learn by observing others [25]. It is the cognitive process that mediates the relationship between social news and behavior [26]. Earlier studies also suggested that emotional states play an important role in the effect of the media [27, 28]. The general learning model (GLM) argues that media exposure affects behaviors by changing individuals’ cognitive, affective or arousal states. In this model, the media content is of great importance in eliciting behavioral responses [29]. Thereby, social news influences what individuals think about and how they feel, and may then influence the information they process, and finally affect how they behave [30]. Hence, social news affects cognition, attitudes, and finally, behaviors [31]. Taken together, if exposure to positive or negative social news affects individuals’ emotional states and cognitive perceptions, then individuals might regulate their cooperative and cheating behaviors.

Based on previous studies and theories above mentioned, this study is unique in that it posited that cues of respect or disrespect for the conventional norm and moral rules delivered in social news would foster further cooperative or cheating behaviors. We call this relationship and influence the “the spreading of social energy.”

## Experiment 1

### Method

#### Participants

Sixty-four undergraduate students (57 females, *M*_*age*_ = 20.61, *SD* = 1.29) participated in the study. They received a small monetary reward for participation, and were told that they also had the chance to earn additional money during the course of the experiment. All of the participants were randomly assigned to one of three conditions: positive, neutral and negative social news videos. The study was approved by the Ethics Committee of the School of South China Normal University. Written, informed consent was obtained from each participant, and all participants were informed of their right to discontinue participation at any time.

#### Stimuli

A total of ten video stimuli were obtained from Chinese websites (e.g. http://paike.youku.com/). Participants were randomly assigned to one of the three experimental conditions (negative, neutral or positive). We used short video clips of 3–5 min duration in each condition, a widely used method used to induce emotion successfully [32]. There were three episodes of harming other people by fighting (i.e., bullying), child abuse (i.e., a nursery teacher beating a child), and dishonest behaviors (i.e., food safety problems) in the negative condition, four episodes that did not involve helping or hurting elements (i.e., Bachelor's Day, football game news, balloon race news and phone snubbing news) in the neutral condition, and three episodes of helping behaviors (i.e., a teacher helping students, citizens helping an unknown patient together, and helping people out of dangerous places) in the positive condition. In each condition, participants watched all the video clips.

To assess whether video clips presented in the procedure are effectively perceived by their corresponding emotions, participants conducted an emotional rating task to these video clips after the experiment. Each participant evaluated the emotions of happy and angry along a 7-point scale from 1 (not at all) to 7 (very much so). Separate one-way ANOVAs were performed on the ratings of happiness and anger using valence (positive, neutral and negative social news) as the independent variable. Results showed that ratings of anger varied across different conditions, *F* (2, 121) = 205.340, *p* < 0.001, η_*p*_^2^ = 0.772. Post-hoc analysis revealed that the mean rating of anger in negative condition (*M* ± *SD* = 5.24 ± 1.32) was significantly higher than that in neutral condition (*M* ± *SD* = 1.42 ± 0.88, *t* (79) = 17.292, *p* < 0.001) and positive condition (*M* ± *SD* = 1.233 ± 0.71, *t* (79) = 18.135, *p* < 0.001). There was no significant difference between positive and neutral conditions on the mean rating of anger (*t* (84) = 0.870, *p* = 0.633). Ratings of happiness also varied across different conditions, *F* (2, 121) = 35.743, *p* < 0.001, η_*p*_^2^ = 0.371. Post-hoc analysis results showed that the mean rating of happiness in positive condition (*M* ± *SD* = 4.47 ± 2.08) was significantly higher than that in neutral condition (*M* ± *SD* = 3.37 ± 1.77, *t* (84) = -3.052, *p* = 0.030) and negative condition (*M* ± *SD* = 1.37 ± 0.75, *t* (79) = -8.377, *p* = 0.003). The mean rating of happiness in negative condition was also significantly lower than that in neutral condition (*t* (79) = -5.420, *p* < 0.001). The ratings of these video clips demonstrated the effectiveness of our emotional manipulation.

#### Design and Procedure

**Fig 1:**
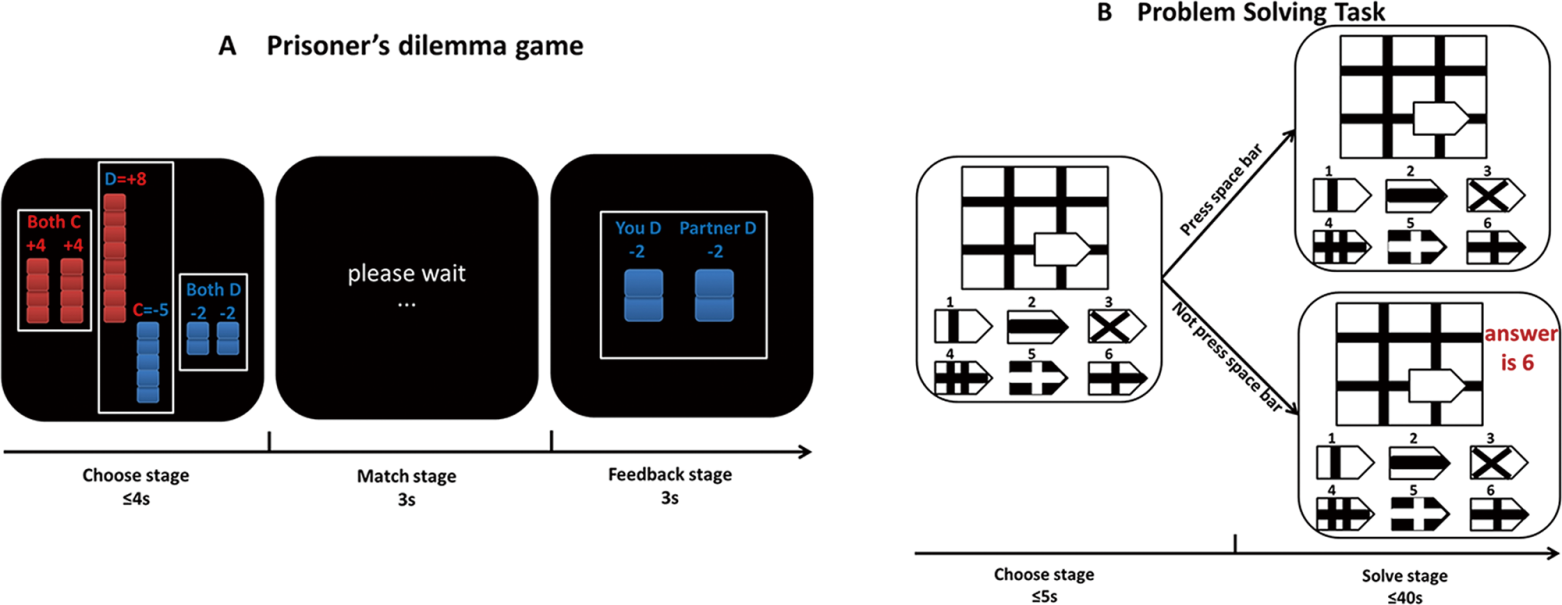
The experimental tasks. **(A). The prisoner’ dilemma game.** In the prisoner’s dilemma game, +8 points to the defector and -5 points to the cooperator when one player defected (D) and the other cooperated (C); +4 points to each player when both players cooperated (C); -2 points to each player when both defected (D). At the beginning of each trial, an asterisk was presented first on the screen for 2s to engage focus and eye fixation. Then the payoff matrix was shown at the center of the screen for 4s, during which participants decided to defect or cooperate, and after that, there was 3s for matching stage. Then feedback was shown for 2s. The player’s choice was presented on the left and the partner’s choice was presented on the right. As participants would earn ¥ 0.2 per point, they were told to try to earn as many points as they could for themselves regardless of others. Participants were informed that they were playing with a same sex partner in another room. **(B). The problem solving task.** At the beginning of each trial, an asterisk was presented first on the screen for 2s to engage focus and eye fixation. On each trial, when problems were presented, the correct answer would appear on the screen unless they stopped it from being shown by pressing the space bar within 5s after the question appeared. Then participants had 40 seconds to answer each question. The task began with four examples so that participants could familiarize themselves with the task.

In this experiment, participants first watched one of the three social news videos. They then played a variant of the prisoner’s dilemma game [33]. The game was performed on a PC using E-Prime software (version 2.0, Psychology Software Tools). There were a total of 12 rounds in the game. Participants were informed that they were playing a strategy game with a same sex partner in another room. Both of them had to choose either cooperation or defection in each round and the outcomes were depended on the two players’ choices. More specifically, +8 points were rewarded to the defector and -5 points were rewarded to the cooperator when one player defected (D) and the other cooperated (C); +4 points were rewarded to each player when both players cooperated (C); -2 points were rewarded to each player when both defected (D) (for the complete instructions, see Fig 1A). Unbeknownst to the participants, they were actually playing against the computer program: the program would defect on the first, fifth, and ninth rounds. We set these invariant responses to make sure that there would be some antisocial responses on the part of the partner, hence preventing the circular pattern of everyone cooperating on each round [33]. On other rounds, the computer was programmed to play tit-for-tat, mimicking the actual player’s response on the preceding round (Fig 1A).

#### Post-experiment task authenticity ratings

Before leaving the lab, participants were instructed to completed a questionnaire with an item inquiring about the possibility that there was an actual partner in the prisoners’ dilemma game (*M* = 4.52, *SD* = 1.69). Answers ranged from 1 (there was no one playing with the participant) to 7 (there was definitely a partner). We conducted this survey in order to make sure that participants believed that this was an interactive game when playing it.

### Results

**Fig 2:**
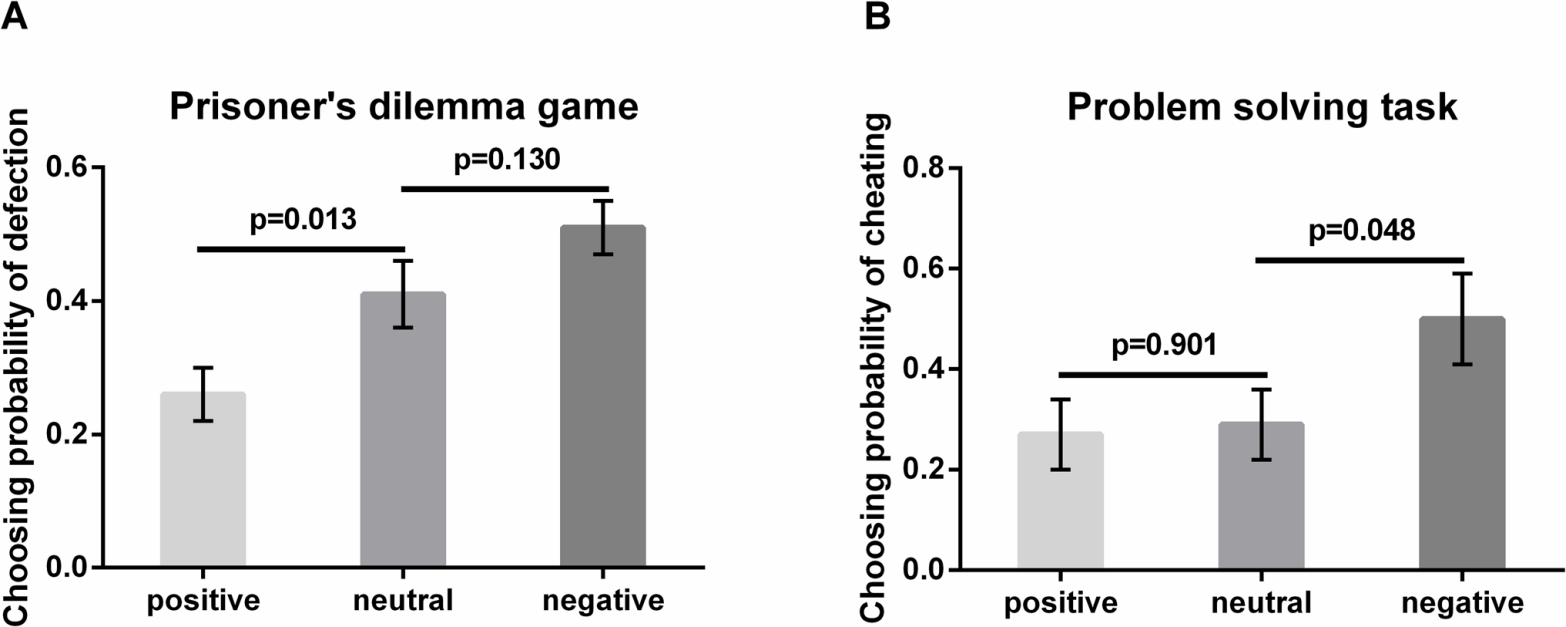
The behavioral results. (A). Choice probability of choosing defection (Experiment 1) in positive, neutral and negative social news conditions. (B). Choice probability of choosing cheating (Experiment 2) in positive, neutral and negative social news conditions. Note: Error bars represent standard errors of the mean.

In experiment 1, we first conducted a one-way ANOVA analysis using valence (positive, neutral and negative social news) as the independent variable and the reaction time as the dependent variable. There were no significant difference among three conditions, *F* (2, 61) < 1, *p* = 0.745, η_*p*_^2^ = 0.010, (negative condition: *M* ± *SD* = 1731 ± 1704 ms; neutral condition: *M* ± *SD* = 1773 ± 1328 ms; positive condition: *M* ± *SD* = 1426 ± 1812 ms). A one-way ANOVA was next performed using valence (positive, negative and neutral social news) as the independent variable and the probability of defection as the dependent variable. The overall results showed that positive social news enhanced the probability of cooperative behaviors (i.e., decreased the probability of defection), as compared to the other two conditions (*F* (2, 61) = 8.343, *p* = 0.001, η_*p*_^2^ = 0.215). Post-hoc analysis revealed that participants in the positive social news group (*M* ± *SD* = 0.258 ± 0.210) showed a lower defection rate compared to both the neutral (*M* ± *SD* = 0.413 ± 0.211, *t* (42) = -2.557, *p* = 0.013, Fig 2A) and negative social news group (*M* ± *SD* = 0.508 ± 0.180, *t* (40) = -4.028, *p* < 0.001, Fig 2A). Although there was a tendency for participants in the negative social news condition to defect more often compared to those in the neutral social news condition, no significant difference was observed (*t* (40) = -1.533, *p* = 0.130, Fig 2A). Because defection behavior decreased in the positive condition, while no significant increase was observed in negative condition, this may suggest that only positive social news affect defection behaviors.

### Discussion

In the prisoners’ dilemma game, defection choices would involve no direct harm or victimization [34], and there is no explicit rule on what individuals should do. Participants could make their decisions freely and both cooperation and defection choices were acceptable. Hence, cooperation choice in this game was more a context-sensitive response but not as a rule that should not be violated [21]. Therefore, rather than moral norm that should be obeyed widely, cooperation behavior in this experiment could be considered to be a conventional norm [35]. The positive social news used in this experiment was about sacrifice and helping others, which primed positive feelings (e.g. happiness). The mood maintenance hypothesis suggests that people experiencing positive moods should be more willing to assist others even at the expense of being taken advantage of in order to sustain the positive mood [36]. Consistent with this assumption, participants in the positive social news group experienced more positive moods and showed higher cooperation even if someone else betrayed them.

Furthermore, helping behavior in the positive social news condition could also indicate a higher level of trust in others. Propensity to trust and cooperation are closely related in human behavior, and studies have demonstrated that the more people trust each other, the more cooperative behavior they will show [37]. In the current study, positive social news about mutual help improved interpersonal trust, thereby increasing the possibility of cooperative behavior. By contrast, negative social news regarding food safety hazards and interpersonal violence contained information about norm-violation, which may arouse feelings of anger and reduce trust, hence reducing the tendency to cooperate. However, the impact of negative social news was not significant in this study, indicating a spreading of positive social energy in the conventional norm domain.

## Experiment 2

Our second study focused on the effect of social news on cheating behavior.

### Method

#### Participants

Sixty-three undergraduate students (54 females; *M*_*age*_ = 20.41, *SD* = 1.33) participated in the study. They received a small monetary reward for participation, and were told that they also had the chance to earn additional money during the course of the experiment. All of the participants were randomly assigned to one of three conditions: positive, neutral and negative social news videos. The study was approved by the Ethics Committee of the School of South China Normal University. Written, informed consent was obtained from each participant, and all participants were informed of their right to discontinue participation at any time.

#### Stimuli

The social news videos used were the same as Experiment 1.

#### Procedure

After the video viewing session, which was identical to that in Experiment 1, participants engaged in a computer task in which they had to choose one out of many simple pictures to make up the whole picture (adapted from Shu and Gino (24)). The stimuli were presented on a PC using E-Prime software (version 2.0, Psychology Software Tools). All 12 problems were multiple-choice questions. Half of these problems were chosen from the hardest difficulty level of Raven's Standard Progressive Matrices [38] and the other six problems were chosen from the hardest difficulty level of a related measure, Raven Progressive Matrices [39]. Each problem had only one correct answer. We informed participants that when they were working on each problem, the correct answer would appear at the top right-hand corner of the participant’s screen unless they stopped it from being shown by pressing the space bar within 5s after the question appeared. Participants needed to pay effort to stop the correct answer from being shown. If participants did press the space bar, then a reaction time would be recorded in this non-cheating trial. This reaction time reflected how long participants take to decide not to cheat. Participants were told that although there were no records of whether or not they had pressed the space bar, they should try to solve these problems on their own. This paradigm cannot tease intentional cheatings and time out cheatings apart. In our study, the mean reaction times in non-cheating trials across the three conditions were shorter than 1.5s (negative condition: *M* ± *SD* = 1481 ± 1026 ms; neutral condition: *M* ± *SD* = 1217 ± 589 ms; positive condition: *M* ± *SD* = 1266 ± 777 ms), suggesting that 5 seconds is adequate for participants to press the space bar to stop the answer from being shown and that majority of participants did not have to rely on cheating in order to achieve the correct answers on most trials. The instructions informed participants that they should solve each problem in 40 seconds and each correct answer will be awarded with ¥ 0.5. Data from debriefing showed that these problems were difficult enough for participants and participants were thus motivated to cheat in order to receive monetary gains. Although participants were informed that space-bar presses were not recorded, in actual, recording these responses was critical to the experimental design, and was therefore implemented without participants’ knowledge. Importantly, the dependent measure of cheating was the percentage of times that participants “forgot” to press the spacebar and thus did not prevent the correct answer from appearing. In this task, cheating occurs not by commission but by omission, and over multiple rounds rather than in one shot. It takes more effort to press the space-bar to prevent the correct answer show up than not press. This procedure ensures that cheating is the dominant response [40–42] (Fig 1B).

#### Post-experiment task difficulty ratings

Before leaving the lab, participants completed a questionnaire that composed of one item inquiring about the difficulty of the problems in the solving task (*M* = 5.16, *SD* = 1.41). Answers varied from 1 (not at all) to 7 (very much so). We conducted this survey to ensure that the task was difficult enough to motivate participants to refer to the answers, thus generating individual cheating behaviors during the problem solving task.

### Results

In experiment 2, we first conducted a one-way ANOVA analysis using valence (positive, neutral and negative) as the independent variable and the reaction times when pressing the space bar in the first five seconds as the dependent variable. Five participants did not press the space bar in all of the trials. We excluded these participants in the following reaction time analyses. There were no differences of reaction times when pressing the space bar among three conditions, *F* (2, 55) < 1, *p* = 0.580, η_*p*_^2^ = 0.02 (negative condition: *M* ± *SD* = 1481 ± 1026 ms; neutral condition: *M* ± *SD* = 1217 ± 589 ms; positive condition: *M* ± *SD* = 1266 ± 777 ms), suggesting the motivation to cheat was equivalent across all social news conditions. We defined cheating as not pressing the spacebar and thus not preventing the correct answer from appearing in the problem solving task. We then conducted a one-way ANOVA analysis using valence (positive, neutral and negative social news) as the independent variable and the probability of cheating as the dependent variable. The probability of cheating on the problem solving task varied with different conditions (***F***(2,60) = 2.888, *p* = 0.063, η_*p*_^2^ = 0.088). Post-hoc analysis revealed that compared to neutral social news (*M* ± *SD* = 0.286 ± 0.307), negative social news enhanced the probability of cheating behavior (*M* ± *SD* = 0.500 ± 0.385, *t* (39) = 2.016, *p* = 0.048, Fig 2B), whereas positive social news did not lead to less cheating behavior (*M* ± *SD* = 0.273 ± 0.327, *t* (41) = 0.125, *p* = 0.901, Fig 2B). Participants in the negative social news condition also showed a significantly higher cheating rate compared to the positive social news condition (*t* (40) = 2.163, *p* = 0.035, Fig 2B). Results indicated a significant increase in cheating behaviors under the influence of negative social news, but not a significant decrease in cheating behaviors under the influence of positive social news. This revealed that only negative social news influenced cheating behaviors.

### Discussion

Cheating is generally believed to be one particular type of moral transgression [43]. In the problem solving task, participants were informed that they should solve the problems on their own–an explicit behavior conduct code. Thus cheating is regarded to be a violation of moral norm [44]. Some participants who did not press the button (i.e., appeared to cheat) reported spontaneously that they forgot the rules during the experiment. This result is consistent with a previous study which suggests that behaving dishonestly leads to the forgetting of rules [24]. Another study demonstrated that social norms displayed by the dishonesty of others and the saliency of dishonesty are vital in individuals’ subsequent unethicality [45]. According to this study, the results here also revealed that negative social news which portrayed unethical behaviors led participants to cheat.

If just watching others’ altruistic or violent behavior changes an individual’s own ethical behavior, how exactly does social news affect a person’s moral conceptions? People often regulate their behavior to be ethical or unethical based on their moral consciousness. Moral self-regulation is a negative feedback process [46]. For example, one study showed that when perceived moral levels were lower than participants’ actual moral levels, the moral licensing effect resulted in reduction of the frequency of ethical behaviors, and when participants perceived higher moral levels, they showed an increase in ethical behavior [47]. In our study, negative social news expressing harm or dishonesty appeared to cause participants to perceive a low moral standard, which resulted in more cheating behavior. Moral levels perceived in the positive condition were likely higher than participants’ own moral standards, and thus participants tended to “purify” their behavior. There is a spreading effect of negative social news in the moral rules domain.

## General Discussion

Earlier research suggested that just watching models behave inappropriately or appropriately can result in more rule infractions or cooperation. For example, some previous studies showed that violent media can increase both violent behavior and decrease prosocial behavior [5], and prosocial media can increase both prosocial behavior and decrease violent behavior [48]. However, contrary to these effects, another study did not find significant correlations between violent game play and cooperation [49]. Consistent with this result, we did not find significant effects of negative social news on conventional behavior, nor effects of positive social news on moral behavior. More specifically, the results we produced here revealed that positive social news increased cooperation but had no effect on cheating behaviors, whereas negative social news generated a significant influence on cheating behavior but had no obvious effect on cooperation. In other words, there was a spreading of social energy which is domain specific. It appears that positive social news mainly affects conventional behaviors (e.g. cooperation) and negative social news mainly influences moral behaviors (e.g. cheating).

Individuals’ behaviors are affected by what they think, believe, and feel [50]. Social cognitive theory states that individuals learn from the impacts of one’s own and others’ actions (e.g. observational learning) [25]. When littering is observed in a given set, it encourages not only littering [51] but also transgressions of other rules like stealing [16]. On the other hand, individuals who just watched other people picking up the dropping empty soda are more willing to help others [17]. As shown in our study, helping behaviors as prosocial cues were observed and thus triggered positive beliefs (e.g. helping). Disobeying moral rules like beating innocent people were perceived as disrespect of moral rules and produced negative views (e.g. violation) and then finally caused the specific effects in cooperation or cheating behaviors.

Compared to social cognitive theory which explains the effects of exposure to media mainly by cognitive priming, the general learning model (GLM) highlights that not only cognition but also emotion and/or arousal could further influence behavioral responses [29]. The GLM predicts that the mental associations activated and formed by social news videos depend on the content of the news [29]. According to GLM, violent or prosocial media (e.g., video games, songs) could increase relative aggressive or prosocial thoughts and ultimately guides behavioral choices [52–58]. Thus it could be argued that self-willing helping in positive social news condition should prime cognitive scripts related to conventional prosocial behaviors, whereas bullying in negative social news was supposed to prime knowledge about moral rules. In addition, the GLM predicts that exposure to media could develop emotional responses which in turn may activate relative behaviors [29]. Earlier research showed that violent media would produce more state hostility and hurtful behaviors yet positive media would induce more positive state affect and helpful behaviors [59]. In our study, consistent with earlier research indicating that media content is strongly associated with emotional states [27], the assessment of emotional states showed that participants felt angrier in negative condition and happier in positive condition compared to the neutral condition. Results then showed more cooperation behaviors in positive condition and more cheating behaviors in negative condition. It seems that the impacts of social news on cooperation and cheating behaviors could work through both the cognitive route and the emotional route.

Taken together, the results suggest that in the positive social news condition, kindness and providing help are the most salient contents–these prime conventional norms mean more altruistic behaviors as well as a greater tolerance for opponents defecting during the prisoner’s dilemma game. In the negative social news condition, harm towards innocent people and unethical behavior are signs of rule violations and lower moral levels. This leads to a greater propensity to break the rules and cheat. In conclusion, this spreading of social energy within a specific domain can be summarized simply as the effect of social cognitive priming. Negative social news mainly affects individual rule behavior but not conventional behavior, whereas positive social news affects perception of conventional norms but not moral rules and thus, influences defection rather than cheating.

The nature of cheating and PDG tasks may also contribute to distinct effects of social news. According to Colman [22], cooperation as an interpersonal behavior mainly involves two persons that each of them only has partial control of the outcomes. Cheating as a personal behavior is mainly determined by the person who decides whether to cheat or not. Thus, whether the task is interpersonal or not may also influence the results. Further studies can examine whether positive social news can facilitate honest moral behavior in interpersonal interactions.

Quality and quantity of social news may also play roles in the spreading effect. We are exposed to the news every day, and not only the quality but also the quantity of this media exposure could perhaps generate a deep influence on human perceptions and behaviors. Further research is warranted to explore the effect of long-term exposure to social news. It should be also noted that this study only examined social events conveyed by mainstream news sources, and no other social media were taken into consideration. Future studies could use a longitudinal design and consider different age groups as well as types of social media. In addition, the exact mechanisms of the spreading effect of social news need to be explored in future research.

The proactive maintenance and violation of social norms in positive and negative social events show a spread of social energy in specific domains. Specifically, positive social news leads to more cooperative behavior but does not result in a significant decrease in cheating behavior. Negative social news leads to more cheating behavior but no noticeable drop in cooperative behavior.
